# Supplementary material for: Kinbank: A global database of kinship terminology
Source: PLoS One. 2023 May 24;18(5):e0283218. doi: 10.1371/journal.pone.0283218 (PMC10208500; doi:10.1371/journal.pone.0283218)
Supplement: S1 File — This file contains additional information on data collection and the methods used in the two examples. (DOCX) [file pone.0283218.s001.docx]

# **Kinbank: Global database of kinship terminology**

Supplementary Material

## Kinbank on Github and Zenodo

Kinbank is the result of collaborative work between the Australian National University, CoEDL (ARC Centre of Excellence for the Dynamics of Language) (Parabank); the University of Bristol (Varikin); the Museu Paraense Emílio Goeldi (MPEGKin); and the University of Helsinki (Kinura). Each dataset is separately ciateble and archived on both Github (for accessibility) and Zenodo (for posterity). The name, citation, and location of each dataset is listed in Table S2.

For each repository, data is stored in raw and cldf format. For the purpose of analyses, we recommend using the cldf dataset. CLDF data is contained within a folder named cldf for each dataset. The CLDF folder contains six files, which are described in table S3. For more detail on the CLDF format see Forkel et al. (2018).

**S1 Table. List of locations and citations for each data collection.**

| **Dataset** | **Archive** | **Github** |
| --- | --- | --- |
| Kinbank | zenodo.org/record/7232746 | github.com/kinbank/kinbank |
| Parabank | zenodo.org/record/7218495 | github.com/kinbank/parabank |
| Varikin | zenodo.org/record/7218606 | github.com/kinbank/varikin |
| MPEGKin | zenodo.org/record/7218387 | github.com/kinbank/goeldi |
| Kinura | zenodo.org/record/7233035 | github.com/kinbank/kinura |
| **Citations** | | |
| Kinbank | Passmore, S., Sheard, C., Barth, W., Quinn, K., Birchall, J., Henrique Oliveria, L., Calladine, J., Ford, M., Argyriou, P., Clifton, I., Deb, A., Harries, L., Hickey-Hall, J., Racz, P., Roberts, S. G., Thomas-Colquhoun, E., Diederen, A., Hoenselaar, L., van den Heuvel, M., … Jordan, F. M. (2023). Kinbank: A global database of kinship terminology. *PLOS ONE, X(X), YYY.* <https://doi.org/10.1371/journal.pone.0283218> | |
| Parabank | Barth, W., Greenhill, S., Quinn, K., Passmore, S., Jordan, F. M., & Evans, N. D. (2022). *Parabank: A global collection of kinship terminology* [Data set]. Zenodo. <https://doi.org/10.5281/zenodo.7218495> | |
| Varikin | Passmore, S., Sheard, C., Argyriou, P., Bowern, C., Calladine, J., Deb, A., Diederen, A., Hickey-Hall, J., Honkola, T., Mitchell, A., Poole, L., Rácz, P., Roberts, S., Ross, R., Thomas-Colquhoun, E., & Jordan, F. M. (2022). *Varikin: A global collection of kinship terminology* [Data set]. Zenodo. <https://doi.org/10.5281/zenodo.7218606> | |
| MPEGKin | Birchall, J., Araujo, L. H., Passmore, S., & Jordan, F. M. (2022). *MPEGKin: A database of kinship terminology from the Tupian and Cariban language families* [Data set]. Zenodo. <https://doi.org/10.5281/zenodo.7218387> | |
| Kinura | Honkola, T., Metsäranta, N., Milanova, V., Passmore, S., & Jordan, F. M. (2022). Kinura: A database of kinship terminology from the Uralic Language family [Data set]. Zenodo. https://doi.org/10.5281/zenodo. 7233035 | |

# **Core kin types**

**S2 Table. Core kin types used for primary search criteria in Kinbank.** If concepts outside this list were available in a language, they were added to the database, but were not the focus of collection. See <https://github.com/kinbank/kinbank/blob/master/kinbank/cldf/parameters.csv> for the full list of concepts.

| *Parameter* | *Description* | *Parameter* | *Description* | *Parameter* | *Description* |
| --- | --- | --- | --- | --- | --- |
| G | sibling | FZD | father's sister's daughter | E | spouse |
| B | brother | FBD | father's brother's daughter | H | husband |
| Z | sister | BD | mother's brother's daughter | W | wife |
| eB | elder brother | ZD | mother's sister's daughter | HF | husband's father |
| yB | younger brother | FBS | father's brother's son | H | husband's mother |
| eZ | elder sister | FZS | father's sister's son | WF | wife's father |
| yZ | younger sister | BS | mother's brother's son | W | wife's mother |
| P | parent | ZS | mother's sister's son | BW | brother's wife |
| F | father | FeBS | father's older brother's son | ZH | sister's husband |
| mM | mother | FyBS | father's younger brother's son | WB | wife's brother |
| C | child | FeZS | father's older sister's son | WZ | wife's sister |
| S | son | FyZS | father's younger sister's son | HB | husband's brother |
| D | daughter | FeBD | father's older brother's daughter | HZ | husband's sister |
| A | ancestor | FyBD | father's younger brother's daughter | co-W | co-wife |
| PP | grandparent | FeZD | father's older sister's daughter | co-H | co-husband |
| FF | father's father | FyZD | father's younger sister's daughter | FW(not) | father's wife (not mother) |
| F | father's mother | eBS | mother's older brother's son | H(notF) | mother's husband (not father) |
| F | mother's father | yBS | mother's younger brother's son | SW | son's wife |
|  | mother's mother | eZS | mother's older sister's son | SW | son's wife's mother |
| CC | grandchild | yZS | mother's younger sister's son | SWF | son's wife's father |
| SS | son's son | eBD | mother's older brother's daughter | DH | daughter's husband |
| SD | son's daughter | yBD | mother's younger brother's daughter | DH | daughter's husband's mother |
| DS | daughter's son | eZD | mother's older sister's daughter | DHF | daughter's husband's father |
| DD | daughter's daughter | yZD | mother's younger sister's daughter | FZH | father's sister's husband |
| FB | father's brother | FBeS | father's brother's older son | FBW | father's brother's wife |
| FZ | father's sister | FByS | father's brother's younger son | ZH | mother's sister's husband |
| B | mother's brother | FZeS | father's sister's older son | BW | mother's brother's wife |
| Z | mother's sister | FZyS | father's sister's younger son |  |  |
| FeB | father's older brother | FBeD | father's brother's older daughter |  |  |
| FyB | father's younger brother | FByD | father's brother's younger daughter |  |  |
| FeZ | father's older sister | FZeD | father's sister's older daughter |  |  |
| FyZ | father's younger sister | FZyD | father's sister's younger daughter |  |  |
| eZ | mother's older sister | BeS | mother's brother's older son |  |  |
| yZ | mother's younger sister | ByS | mother's brother's younger son |  |  |
| eB | mother's older brother | ZeS | mother's sister's older son |  |  |
| yB | mother's younger brother | ZyS | mother's sister's younger son |  |  |
| BS | brother's son | BeD | mother's brother's older daughter |  |  |
| BD | brother's daughter | ByD | mother's brother's younger daughter |  |  |
| ZS | sister's son | ZeD | mother's sister's older daughter |  |  |
| ZD | sister's daughter | ZyD | mother's sister's younger daughter |  |  |
| eBS | older brother's son |  |  |  |  |
| yBS | younger brother's son |  |  |  |  |
| eBD | older brother's daughter |  |  |  |  |
| yBD | younger brother's daughter |  |  |  |  |
| eZS | older sister's son |  |  |  |  |
| yZS | younger sister's son |  |  |  |  |
| eZD | older sister's daughter |  |  |  |  |
| yZD | younger sister's daughter |  |  |  |  |

**S3 Table. CLDF table descriptions of Kinbank.**

| File | Description |
| --- | --- |
| cldf-metadata.json | This is a metadata description file, which contains a json description of each file in the cldf folder, and each column within those files to ensure the data is formatted correctly. |
| forms.csv | This contains the forms, or kinterms, for each language. This file links to the languages file by the Languages_ID column, parameters file by the Parameters_ID column, and the sources file by the sources_bibtex column. |
| languages.csv | This file contains metadata on each language including: glottocode, geographic coordinates, ISO639P3 code, and Language family. |
| parameters.csv | This file contains descriptions of each kin type used in the dataset. |
| Sources.bib | A bibtex file containing information on the sources used in Kinbank. |
| requirements.txt | This file contains the python package requirements for installing Kinbank |

# **Kinbank Inter-rater reliability**

Forty-four languages were collected in both Parabank and Varikin, and these are used to determine the level of intercoder reliability. A major avenue for error is when kinship terms are collected for one language but two different sources across the projects which disagree on kin terms. Of most interest is ensuring the structural paradigm of a particular kinship terminology is consistent (i.e. that all *parent's female siblings* are syncretised to "aunt" in English). This is what is used to determine inter-coder reliability. To test for structural similarity, all pairs of syncretised kin types within each language are identified in each collection. This process creates a binarised vector for each language in each database, representing the structure of each language. This vector is used to compare whether the presence or absence of syncretisms are the same across collections in the 44 languages. The sum of matches divided by the total number of pairs gives a measure of structural similarity, where a score of 1 will indicate an exact structural match. Across all compared relationships, this gives us a structural similarity value of 0.80. Taking a random sample of compared relationships gives us a mean value of 0.74 (Table S4).

This score is calculated for various subsets of kin types to check for any focused areas of differences. Amongst the subsets there are high levels of agreement amongst parents, children, parent's parents, parent's sibling's children (from 0.89 to 0.74; Table S3), but lower levels of agreement between siblings and sibling's children.

**S4 Table. Structural similarity scores between Parabank and VariKin collections in a set of 44 overlapping languages.** The average similarity of the total structure of languages is 0.80, and with a random sample 0.74. If languages are coded identically, the structural similarity will have a score of 1, and if they are complete opposites, they will have a score of 0. Italicised rows indicate similarity scores that consider the entire sample.

| Relationship subset | Structural similarity |
| --- | --- |
| *Complete system* | *0.80* |
| *Random sample* | *0.74* |
| Parents | 0.89 |
| Children | 0.83 |
| Parent's parents | 0.79 |
| Parent's sibling's children | 0.74 |
| Affines | 0.69 |
| Parent's siblings | 0.68 |
| Children's children | 0.64 |
| Parent's Parent's siblings | 0.62 |
| Siblings | 0.59 |
| Sibling's children | 0.42 |

These results give confidence that Kinbank shows reliable structural similarity. Other proposed errors of kinship terminology transcription include orthographic errors, transcription errors, misinformation, within-language variability, and linguistic change not accounted for here.

## Example 1: Are you my mama? Nursery words and sound meaning

## Model summary

We implement a repeat-measures phylogenetic Bayesian logistic regression model, following the implementation laid out in Bürkner (2021). This model contains a binary response indicating whether the kinterm refers to a mother (1) or father (0). We have a minimum of two words per language, which we treat as repeated measurements for statistical purposes. This model contains two fixed effects, and two random effects. The fixed effects are different categories of vowel or consonant sounds, laid out in Blasi et. al (2018) and as mentioned in the main text. One random effect contains the variance-covariance matrix for a global phylogeny, representing the relationships between languages on the global scale Jäger (2018). We include a second random effect, that is independently and identically distributed, to capture variance between languages that might represent non-phylogenetic influence. Results of the model are displayed below in Table S5.

**S5 Table. Table of coefficients for the phylogenetically-controlled repeated measures multi-level Bayesian logistic regression.** Significant coefficients are in bold.

|  | Estimate | Est.Error | lower 95% CI | Upper 95% CI | Rhat | Bulk_ESS | Tail_ESS |
| --- | --- | --- | --- | --- | --- | --- | --- |
| Intercept | 0.32 | 0.92 | -1.46 | 2.17 | 1 | 2049 | 3434 |
| vowel3 | -0.35 | 1.1 | -2.53 | 1.78 | 1 | 3124 | 5134 |
| vowela | -0.42 | 0.86 | -2.15 | 1.24 | 1 | 2207 | 3287 |
| vowele | 0.43 | 0.87 | -1.31 | 2.13 | 1 | 2263 | 3515 |
| vowelE | 1.01 | 1.01 | -1 | 2.98 | 1 | 2718 | 4667 |
| voweli | 1.12 | 0.86 | -0.61 | 2.79 | 1 | 2233 | 3506 |
| vowelo | -0.8 | 0.88 | -2.57 | 0.88 | 1 | 2266 | 3418 |
| vowelu | -0.07 | 0.87 | -1.81 | 1.63 | 1 | 2248 | 3459 |
| consonant5 | 0.07 | 1.77 | -3.57 | 3.64 | 1 | 12165 | 7997 |
| consonant7 | -0.32 | 0.61 | -1.54 | 0.86 | 1 | 4112 | 6629 |
| consonant8 | -1.89 | 1.39 | -5.06 | 0.44 | 1 | 7657 | 6294 |
| **consonantb** | **-1.64** | **0.36** | **-2.35** | **-0.94** | **1** | **1706** | **3662** |
| consonantc | -1.28 | 0.8 | -2.87 | 0.27 | 1 | 5953 | 7578 |
| consonantC | -1.01 | 0.54 | -2.09 | 0.01 | 1 | 3326 | 5652 |
| consonantd | -0.44 | 0.39 | -1.21 | 0.32 | 1 | 2017 | 4102 |
| **consonantf** | **-1.31** | **0.51** | **-2.31** | **-0.33** | **1** | **3150** | **6267** |
| consonantg | 0.13 | 0.46 | -0.8 | 1.03 | 1 | 2593 | 5222 |
| consonantG | 2.2 | 1.38 | -0.1 | 5.38 | 1 | 9746 | 7001 |
| consonanth | -0.52 | 0.43 | -1.36 | 0.33 | 1 | 2380 | 4925 |
| consonantj | 0.3 | 0.48 | -0.63 | 1.26 | 1 | 2850 | 5042 |
| consonantk | 0.13 | 0.36 | -0.58 | 0.83 | 1 | 1654 | 3513 |
| consonantl | -0.2 | 0.46 | -1.11 | 0.68 | 1 | 2655 | 5284 |
| consonantm | 0.09 | 0.32 | -0.53 | 0.72 | 1 | 1430 | 2658 |
| **consonantn** | **1.03** | **0.34** | **0.37** | **1.7** | **1** | **1578** | **3270** |
| **consonantN** | **1.44** | **0.37** | **0.71** | **2.16** | **1** | **1728** | **3659** |
| **consonantp** | **-2.02** | **0.36** | **-2.73** | **-1.34** | **1** | **1702** | **3661** |
| **consonantr** | **-0.91** | **0.45** | **-1.79** | **-0.02** | **1** | **2615** | **4956** |
| **consonants** | **-1.1** | **0.38** | **-1.85** | **-0.37** | **1** | **1868** | **4011** |
| consonantS | -0.77 | 0.97 | -2.68 | 1.16 | 1 | 8193 | 8284 |
| **consonantt** | **-1.69** | **0.34** | **-2.34** | **-1.03** | **1** | **1565** | **3008** |
| consonantv | 0.03 | 0.52 | -0.97 | 1.07 | 1 | 3204 | 6087 |
| consonantw | 0.57 | 0.41 | -0.24 | 1.36 | 1 | 2176 | 4513 |
| consonantx | -8.22 | 6 | -22.63 | 0.43 | 1 | 10224 | 6969 |
| consonantX | 0.83 | 1.02 | -1.06 | 2.95 | 1 | 7476 | 7187 |
| consonanty | -0.11 | 0.36 | -0.83 | 0.6 | 1 | 1708 | 3520 |
| consonantz | -1.58 | 1.04 | -3.75 | 0.38 | 1 | 9006 | 7707 |

**S6 Table. Description of vowel and consonant codes.** Refer to S1 table in Blasi et al. (2016).

| **Symbol** | **Description** |
| --- | --- |
| p | Voiceless bilabial stop and fricative |
| b | Voiced labial stop and fricative |
| m | Bilabial nasal |
| f | Voiceless labiodental fricative |
| v | Voiced labiodental fricative |
| 8 | Voiceless and voiced dental fricative |
| 4 | Dental nasal |
| t | Voiceless alveolar stop |
| d | Voiced alveolar stop |
| s | Voiceless alveolar fricative |
| z | Voiced alveolar fricative |
| c | Voiceless and voiced alveolar fricative |
| n | Voiceless and voiced alveolar nasal |
| S | Voiceless postalveolar fricative |
| Z | Voiced postalveolar fricative |
| C | Voiceless palato-alveolar affricate |
| j | Voiced palato-alveolar affricate |
| T | Voiceless and voiced palatal stop |
| 5 | Palatal nasal |
| k | Voiceless velar stop |
| g | Voiced velar stop |
| x | Voiceless and voiced velar fricative |
| N | Velar nasal |
| q | Voiceless and voiced uvular stop |
| X | Voiceless and voiced uvular fricative, voiceless and voiced pharyngeal fricative |
| 7 | Voiceless glottal stop |
| h | Voiceless and voiced glottal fricative |
| l | Voiced alveolar lateral approximant |
| L | All other laterals |
| w | Voiced bilabial-velar approximant |
| y | Palatal approximant |
| r | All varieties of “r sounds” |
| i | High front vowel, rounded and unrounded |
| e | Mid front vowel, rounded and unrounded |
| E | Low front vowel, rounded and unrounded |
| 3 | High and mid central vowel, rounded and unrounded |
| a | Low central vowel, unrounded |
| u | High back vowel, rounded and unrounded |
| o | Mid and low back vowel, rounded and unrounded |

## Interrater reliability of parental word codings

Parental words were coded entirely by SP. To check the validity of codings between 120-180 parental terms (6% of the sample) were coded by three other authors (PR, AD, JC). We compare codes between all pairs of authors, using Cohen’s Kappa, and between multiple coders simultaneously using Light’s Kappa. Codes for consonants and vowels are compared separately.

Using Kappa value interpretation guidelines from Landis & Koch (1977), we have moderate vowel agreement and substantial consonant agreement across all raters. The lower vowel agreement is primarily attributed to coders having different understandings of low central vowel sounds and low front vowel sounds. Between the two more experienced coders (PR and SP) there is a higher level of agreement.

**S7 Table. Interrater reliability statistics.** Between pairs of coders Cohen’s kappa is used, and between all coders, Light’s Kappa. Statistics can be interpreted using guidelines from Landis & Koch (1977): <0 No agreement, 0 — .20 Slight, .21 — .40 Fair, .41 — .60 Moderate, .61 — .80 Substantial, .81–1.0 Perfect.

| **Coder 1** | **Coder 2** | **Vowel** | **Consonant** | **N** |
| --- | --- | --- | --- | --- |
| *AD* | *JC* | 0.35 | 0.81 | 104 |
| *AD* | *PR* | 0.78 | 0.66 | 44 |
| *AD* | *SP* | 0.59 | 0.78 | 114 |
| *JC* | *PR* | 0.40 | 0.80 | 44 |
| *JC* | *SP* | 0.39 | 0.91 | 110 |
| *PR* | *SP* | 0.89 | 0.72 | 95 |
| *All* | *All* | 0.60 | 0.79 |  |

# **Example 2: Does crossness indicate marriage preferences?**

## Coding of Bifurcate merging terminology

Coding for the presence of bifurcate merging terminology was determined by the presence of each of these rules (when referring to a specific gender) or both rules:

Father = Father’s Brother ≠ Mother’s Brother

Mother = Mother’s Sister ≠ Father’s Sister

This pattern is often strictly followed; however, it is common in sub-Saharan Africa for parent's same-sex siblings to be terminologically equivalent but distinguished by age through terms such as "big" and "little" parent. Languages making these terminological distinctions are also coded as instances of bifurcate merging, provided there is a distinct MB term. Coding for these patterns by gender created three binary variables: presence of bifurcate merging in men, in women, and complete bifurcate merging. Although there was no pre-existing theoretical reason for making the male / female distinction it became clear while coding that merging patterns are not always symmetrical across sexes, so they are conservatively analysed both separately and together.

## Cross-cousin marriage data

Data on allowable cross-cousin marriage is taken from the Ethnographic atlas question *EA023: Cousin marriage permitted*. This variable contains thirteen categories of allowable cousin-marriage (including no cousin marriage), eight of which relate to various forms of cross-cousin marriage, from which we derive an allowable cross-cousin marriage binary variable (EA023 codes: 1-6, 9, and 11, see table S4 for a coding table). In summary, there are three analyses performed between one cousin marriage variable, and three alternative codings of bifurcate merging terminology.

## Bifurcate merging data

This table contains data on the presence/absence of bifurcate merging in each language used in the analysis, their taxon code used in the Grollemund et. al (2015) phylogeny. A society is coded as practising cross-cousin marriage if: a cross-cousin is a possible marriage partner and all parallel cousins are not marriageable. This means that not all cross-cousins must be marriageable for us to code cross-cousin marriage as present.

**S8 Table. Bifurcate-merging (BM) codes, derived from Kinbank.** Codes for the 61 Bantu languages, alongside their taxa codes used in the Grollemund et al. (2015) phylogeny.  1 indicates presence of bifurcate merging, 0 indicates an absence, and -1 is no data.

| Name | Taxon | Male BM | Female BM | Complete BM |
| --- | --- | --- | --- | --- |
| Bakweri | A22_Bakweri | 1 | 0 | 0 |
| Batanga | A32C_Batanga | 1 | 1 | 1 |
| Fang Bitam | A75a_Fang_Bitam | 1 | 1 | 1 |
| Kota | B25_Kota | 1 | 1 | 1 |
| Mbunda | B84_Mbunda | 1 | 1 | 1 |
| Yanzi | B85_Yanzi | 0 | 0 | 0 |
| Nsongo | B85d_Nsongo | 1 | 1 | 1 |
| Dinga | B86_Dinga | 1 | 1 | 1 |
| Bushong | C61E_Konda | 1 | 1 | 1 |
| Songola | C71_Tetela | 1 | 1 | 1 |
| Kikuyu | C83_Bushong | 1 | 1 | 1 |
| Meru | D24_Songola | 1 | 0 | 0 |
| Digo | E51_Kikuyu | 1 | 0 | 0 |
| Bende | E53_Meru | 1 | 1 | 1 |
| Giryama | E72a_Giryama | 1 | 1 | 1 |
| Digo | E73_Digo | 1 | 1 | 1 |
| Bende | F12_Bende | 1 | 1 | 1 |
| Sukuma | F21_Sukuma | 1 | 1 | 1 |
| Fefe Grassfields | Fefe_Grassfields | 0 | 0 | 0 |
| Gogo | G11_Gogo | 1 | 1 | 1 |
| Kagulu | G12_Kagulu | 0 | 0 | 0 |
| Kwere | G32_Kwere | 1 | 1 | 1 |
| Nguungulu | G34_Nguungulu | -1 | 1 | 0 |
| Luguru | G35_Luguru | 1 | 1 | 1 |
| Sangu | G61_Sangu | 1 | 1 | 1 |
| Hehe | G62_Hehe | 1 | 1 | 1 |
| Bena | G63_Bena | 1 | 1 | 1 |
| Kisikongo (2013) | H16a_Kisikongo_2013 | 1 | -1 | 0 |
| Yombe | H16c_Yombe | 1 | 1 | 1 |
| Suku | H32_Suku | 1 | 1 | 1 |
| Mbala | H41_Mbala | 1 | 1 | 1 |
| Shi | JD53_Shi | 1 | 1 | 1 |
| Rundi | JD62_Rundi | 0 | 0 | 0 |
| Kiha | JD66_Kiha | 1 | 1 | 1 |
| Runyoro | JE11_Runyoro | 1 | 1 | 1 |
| Luganda | JE15_Luganda | 1 | 1 | 1 |
| Lusoga | JE16_Lusoga | 0 | 1 | 0 |
| Haya | JE22_Haya | 0 | 1 | 0 |
| Kerebe | JE24_Kerebe | 1 | 1 | 1 |
| Bukusu | JE31c_Bukusu | 1 | 1 | 1 |
| Gusii | JE42_Gusii | 1 | -1 | 0 |
| Ciokwe | K11_Ciokwe | 1 | 1 | 1 |
| Lwena | K14_Lwena | 1 | 1 | 1 |
| Lozi | K21_Lozi | 0 | 0 | 0 |
| Pende | L11_Pende | 1 | 1 | 1 |
| Luba-Kasai | L31a_Luba-Kasai | 1 | 1 | 1 |
| Sanga | L35_Sanga | 1 | -1 | 0 |
| Kaonde | L41_Kaonde | 1 | 1 | 1 |
| Lunda | L52_Lunda | 1 | 1 | 1 |
| Fipa | M13_Fipa | 1 | 1 | 1 |
| Bemba | M42_Bemba | 1 | 1 | 1 |
| Lamba | M54_Lamba | 1 | 1 | 1 |
| Tonga | M64_Tonga | 1 | 0 | 0 |
| Ngoni | N12_Ngoni | 1 | 1 | 1 |
| Simakonde | P23_Simakonde | 1 | 1 | 1 |
| Umbundu | R11_Umbundu | 1 | 1 | 1 |
| Herero | R31_Herero | 0 | 1 | 0 |
| Venda | S21_Venda | 1 | 1 | 1 |
| Tswana | S31_Tswana | 1 | 1 | 1 |
| Zulu | S42_Zulu | 1 | 1 | 1 |
| Tsonga | S53_Tsonga | 1 | 1 | 1 |

**S9 Table. Coding for Cousin marriage statistics.** Presence (1) or absence (0) or permitted cross-cousin marriage using EA023 from the Ethnographic Atlas (Murdock, 1967; Kirby et al., 2016)

| Code | Type of marriage | Coding |
| --- | --- | --- |
| 1 | Duolateral cross-cousin marriage permitted, i.e., marriage allowed with either MoBrDa or FaSiDa but forbidden with a parallel cousin | 1 |
| 2 | Duolateral marriage permitted with paternal cousins only (FaBrDa or FaSiDa) | 0 |
| 3 | Duolateral marriage permitted with maternal cousins only (MoBrDa or MoSiDa) | 0 |
| 4 | Duolateral marriage permitted with an uncle's daughter only (FaBrDa or MoBrDa) | 0 |
| 5 | Duolateral marriage permitted with an aunt's daughter only (FaSiDa or MoSiDa) | 0 |
| 6 | Unilateral: only matrilateral cross-cousin marriage permitted, i.e., with a MoBrDa | 1 |
| 7 | Nonlateral marriage, i.e., unions forbidden with any first or second cousin | 0 |
| 8 | Nonlateral marriage, evidence available only for first cousins | 0 |
| 9 | Unilateral: only patrilateral cross-cousin marriage permitted i.e., with a FaSiDa | 1 |
| 10 | Quadrilateral marriage, i.e., marriage allowed with any first cousin | 0 |
| 11 | Nonlateral marriage in which all first cousins and some but not all second cousins are forbidden as spouses | 0 |
| 12 | Nonlateral marriage in which unions are forbidden with any first cousin but are permitted with any second cousin (or at least any who is not a lineage mate) | 0 |
| 13 | Trilateral marriage, i.e., marriage allowed with any first cousin except an ortho-cousin or lineage mate | 0 |

## MCMC trace plots

**S10 Table. Gelman Rubin Diagnostic statistics.** Statistics are shown for the independent and dependent model of each analysis. Showing the Estimate and, in brackets, the Upper 95% confidence interval. Gelman and Rubin statistic <1.1 are shown to have converged.

| ***Model*** | ***Independent*** | ***Dependent*** |
| --- | --- | --- |
| Male Bifurcate Merging | 1 (1) | 1 (1) |
| Female Bifurcate Merging | 1 (1) | 1 (1.01) |
| Complete Bifurcate Merging | 1 (1) | 1 (1) |


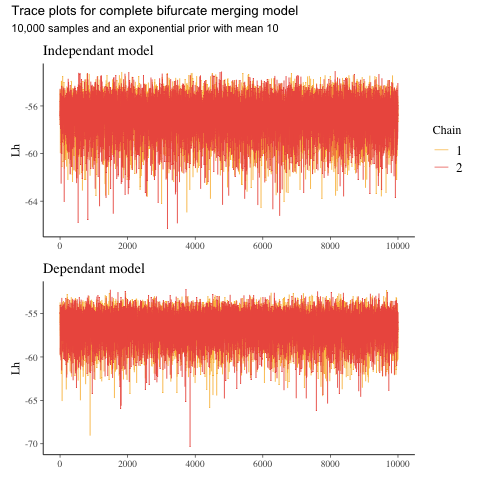

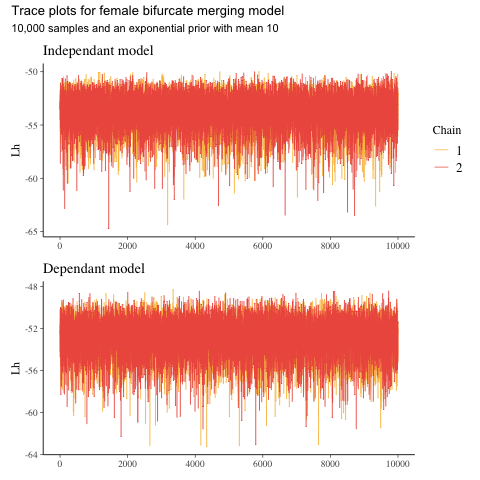

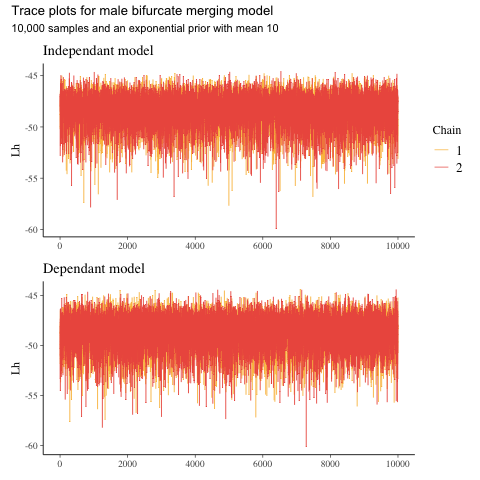


**S1 Fig**. **Bayesian Model convergence plots.** Plots show MCMC results for each of the three co-evolutionary models (male bifurcate merging, female bifurcate merging, and complete bifurcate merging). Each model is a sample of 10,000 iterations over two chains. All models are seen to converge.

# **References**

Blasi, D. E., Wichmann, S., Hammarström, H., Stadler, P. F., & Christiansen, M. H. (2016). Sound–meaning association biases evidenced across thousands of languages. Proceedings of the National Academy of Sciences, 113(39), 10818–10823. https://doi.org/10.1073/pnas.1605782113

Bürkner, P.-C. (2021). Estimating Phylogenetic Multilevel Models with brms. <https://cran.r-project.org/web/packages/brms/vignettes/brms_phylogenetics.html>

Forkel, R., List, J.-M., Greenhill, S. J., Rzymski, C., Bank, S., Cysouw, M., Hammarström, H., Haspelmath, M., Kaiping, G. A., & Gray, R. D. (2018). Cross-Linguistic Data Formats, advancing data sharing and re-use in comparative linguistics. Scientific Data, 5(1), 1–10. <https://doi.org/10.1038/sdata.2018.205>

Jäger, G. (2018). Global-scale phylogenetic linguistic inference from lexical resources. Scientific Data, 5(1), 180189. https://doi.org/10.1038/sdata.2018.189
