# Supplementary material for: Kinbank: A global database of kinship terminology
Source: PLoS One. 2023 May 24;18(5):e0283218. doi: 10.1371/journal.pone.0283218 (PMC10208500; doi:10.1371/journal.pone.0283218)
Supplement: Attachment — Submitted filename: Response to Reviewers.docx [file pone.0283218.s002.docx]

Below is a point-by-point response to each reviewer’s comments and concerns, reviewer comments are in red.

Sam Passmore, Nicholas Evans, and Fiona Jordan (on behalf of all authors).

*Reviewer #1*

This will be a very useful dataset for many social scientists. I wish I had access to such a dataset years ago. I believe the paper meets the standards of the journal. The dataset is constructed carefully and kinterms make sense when I checked them for the few languages which I am familiar with.

However, I have a few small comments and suggestions:

My main concern is that the sample of 1,168 languages (out of 7,560 live languages according to the Ethnologue) might not be a representative sample of the world population. For example, if you are relying on the politically dominant languages of countries (which are more likely to have dictionaries), there will be a selection bias in your sample. The abstract says “representative sample”, but there is no discussion of that in the paper. What percentage of world population and each continent is covered by the sample? You can use Ethnologue maps and population data to give us some estimates and convince us that this is indeed a representative sample. It would be helpful if you tell us why thousands of live languages are no included in the sample. Not all readers are familiar with your data collection restrictions.

First, we thank the reviewer for thoughtful comments that we are sure will improve the impact of this database and paper. On reflection, the use of ‘representative’ is misleading (though is our ultimate ambition) and we have removed it. However, we do emphasise that this dataset is the largest collection of kinship terminology to date. We have added additional information to the section on data collection and sampling to highlight more of the strengths (e.g. phylogenetic coverage) and weaknesses of our approach. It is likely that, on aggregate, Kinbank does contain sampling biases, but this is also the case for all existing large-scale cultural databases. The virtue of these new cultural database projects (including ours), is that they are much larger and more diverse than their predecessors. We have changed the abstract wording on line 38 to the following:

*Here we present Kinbank, a new database of 210,903 kinterms from a global sample of 1,229 spoken languages.*

We added the following text on line 218 describing the collection procedure in more detail:

*Parabank collected kinship terminologies opportunistically, including the digitisation of the terminologies from Morgan’s (1871) landmark global kinship survey, Systems of Consanguinity and Affinity. The global nature of the Parabank sample means the database holds terminologies from societies with a range of ecological pressures and kinship structures, allowing us to explore convergent patterns of terminology (e.g. Passmore et al., 2021) across the global sample.*

*Varikin focused on sampling languages that are paired both with a dated language phylogeny (e.g. Gray et al., 2009) and with existing anthropological databases (e.g. D-PLACE; K. R. Kirby et al., 2016). The most sampled language families by Varikin are Austronesian (n = 375), Atlantic-Congo (117), Indo-European (105), and Pama-Nyungan (104). This sampling strategy allows for a range of phylogenetic analyses that control for patterns of autocorrelation that might occur through descent (Jordan & Dunn, 2010, Roberts & Winters 2013). The focus on societies within the Ethnographic Atlas additionally allows kinship terminology data to be connected to Demographic and Health surveys (Bahrami-Rad, Becker, Henrich, 2021).*

*MPEGKin compiled data on two large language families of South America, Tupian and Cariban, sampling as densely as possible across the major branches of the families given the available ethnographic and linguistic documentation. Beyond making new data available for these lesser-known languages (Birchall & Jordan 2019), MPEGKin was designed to support quantitative comparative studies and traditional linguistic reconstruction with these language families (e.g., Birchall et al. 2019, Carvalho & Birchall 2022).*

*Kinura collected kinship terminologies of all the main groups of the Uralic family. The sampling focused on matching the kin term data with the speaker groups included in the genetic study of Tambets et al. (2018).*

The goal of Kinbank was to offer a sample of global linguistic diversity, rather than population diversity. We have added the following text to line 81:

*With Kinbank, we centralise terminology from approximately 15% of known language diversity, at least 3.28 billion speakers (47% of the speakers in Ethnologue), including speakers for 96% of the territories covered in Ethnologue, and at least 20% of every continent. Kinbank contains the largest and broadest collection of terminology available, offering a platform to investigate variation in kinship terminology.*

It would be interesting to see example 2 presented with cousin marriage preference variable (EA025) as well. Some argue that cousin marriage preference better reflects the actual marriage practices of societies than cousin marriage permitted. According to Jack Goody (1976)’s analyses, there is a strong correlation between inheritance, cousin marriage, and Eskimo and descriptive categories of cousin terms. I am not aware of later literature on Goody’s hypothesis or analyses, but it might worth considering it as an additional example.

We thank the reviewer for this suggestion. The reason we chose cousin marriage permitted (over preference) was to test a hypothesis posited by Viveiros de Castro (1998) which suggested that cousin terms would display crossness in the absence of preferred cousin marriage because cousin marriage was a historical practice. That is, the kin terms reflect the historical marriage tradition. We might reasonably expect that permitted cousin marriage to be the behavioural state of a society that is moving away from cousin marriage and therefore still predict crossness in cousin terms.

Since our hypothesis focused on crossed cousin terms, and not Linear (equivalent to what has historically been called "Eskimo") or Descriptive types, we feel that extending the analysis to the reviewer's suggestion begins to stretch the purpose of this example. This example is intended to inspire more studies, like the one suggested. We hope our example will lead researchers to investigate these issues further with regard to Lineal, Descriptive types or any other terminological structure and other behaviours. An additional reference in the same vein is Goody (1970), which contains numerous correlations between kinship terminology and kinship behaviour, which we believe need re-testing within a phylogenetic framework and with more granularity in kin term structure.

Choosing appropriate examples to showcase a database is a trade-off between the rich literature behind each possibility and the need to keep the examples themselves clear and relevant. We have added the following to line 623 of the conclusion of the second example to encourage future research:

*However, we test only one example of a behavioural-linguistic link from a sea of proposed hypotheses (e.g. Goody 1970, 1976), and we hope this database encourages future research on the relationship between kinship norms and language.*

It might be useful to mention that your dataset can be linked to today’s many surveys. Giuliano & Nunn (2018) and Bahrami-Rad, Becker, Henrich (2021) provide methods to link EA to survey data. The later provides the data on a website: <http://dgce.fas.harvard.edu/>. Despite its all shortcoming, the method seems to be becoming popular among economic historians.

This is a good suggestion to increase the audience of the database. We have added the following text to the manuscript to show the applicability of kinship terminology to economic historians and how the database could be linked to databases they are interested in.

On line 132 of the introduction, we point more directly to economic historians focus on kinship:

*Kinship is also becoming increasingly central to economic historians, who have used kinship terminology structure to infer broader patterns of social structure (Henrich 2021, Schulz 2021).*

On line 252 in the section *The Kinbank Sample*, we point to the link to survey data:

*The focus on societies within the Ethnographic Atlas additionally allows kinship terminology data to be connected to Demographic and Health surveys (Bahrami-Rad, Becker, Henrich, 2021).*

*Reviewer #2*

In this article, the authors introduce a new database of the diversity of terms with which speakers of a large number of languages refer to their genealogical kin. After a short introduction, they present two example analyses for the potential that this database might offer for future research into the links between language, cognition, and behaviour.

Thank you for the helpful reflections on how our database may be received by our various intended audiences, as well as for the positive tone. We have added several clarifying points throughout, the most important of which we detail below.

My main problem with the article in the current version is that I don't think there is sufficient information on how the database was built, which supposedly is the key point of this article.

The reviewer made a similar comment that we address at the same time:

How was the database actually built? Is the information all from secondary literature, or did it involve primary data collection for some of the languages of anthropologists asking native speakers about the terms and their meaning? If it is based on secondary literature, how do you account for the potential risk that original encoders might have had a particular focus (either because of their research focus or their own cultural background) such that they might not have recorded all relevant kin terms?

The reviewer is right to ask for more information on the building of the database. In response to the above reviewer, we have added additional information on how languages were sampled across the different projects. In addition, we have added the following section *Collection Procedure* on line 347 which describes the collection procedure used across projects:

*Data are entered into Kinbank as either primary or secondary data, and is affiliated with a source reference detailing where the data came from. The data are most commonly secondary, and transcribed by coders into the Kinbank collection template. Secondary sources ranged from ethnographies and grammars, to simpler descriptions like wordlists. In the majority of cases, secondary sources describe kin terms etically following the tradition laid out by Morgan (1871). The etic descriptions usually align with the list of 155 core kin types. In these instances, kin term collection is a matter of copying terms to the appropriate place. It is common for source categories to be less refined than Kinbank kin types. In this case, coders would apply the term to all relevant kin types. For example: the kin type father’s brother is commonly used in secondary sources, but this aggregates four Kinbank kin types (father’s elder brother, father’s younger brother, and whether the speaker is male or female). A coder would apply the term to all four of these kin types in the absence of further information. In the rare instance that kin terms were not etically described (often using English nomenclature), coders would search the text for context and confirmation that these categories align, and record any inferences in the notes column for each kin term. Coders were also encouraged to flag where original sources may have errors.*

*Kin terms are transcribed as closely as possible to their source form. Since terminologies are predominantly collected by anthropologists and not linguists, sources are primarily in Roman script (as opposed to other standardized orthographies) and can contain transcription inconsistencies across languages. Kinbank makes no judgments on which transcriptions are correct or incorrect, besides obvious typos. The database accommodates phonetic transcriptions in the International Phonetic Alphabet where these are available. Some sources recorded terminology using Cyrillic; in this case kin terms are transliterated into Roman characters, with the original form in an adjacent column. This approach means that the copying is consistent within source but may not be consistent across sources or languages.*

*Some data was elicited from native speakers or academics actively working with a particular language. In this case the person(s) who offered the terms is listed as the source. When data was collected from native speakers, the fieldworker would use various methods to collect kin terms. For example: the fieldworker would have informants explain their genealogy, eliciting kin terms through this process, coupled with participant observation to observe the kinterms being used in context. Academics actively studying a language were provided with the template sheet and a description of the task and asked to fill in the categories with any necessary comments or adjustments, using the methods above. When there was specific context, meaning, or otherwise interesting information for a particular kinterm it was recorded in the notes. Some sources (speakers or secondary) may offer many more kinterms that apply beyond the 155 core types. In this instance, we expand the parameter list to accommodate the additional kin terms (see table S2). In total there are 940 kin types within Kinbank, but many of these are sparsely populated. Some languages also contain multiple terms for a particular category. In this instance each kin term is entered separately.*

For example, it seems like you currently would not advise an analysis into why certain terms are missing from specific languages?

This would be an oversimplification. In fact, we would encourage users of the database to explore why terms are missing in specific languages and offer corrections should they find errors. We do advise that assuming missing items to be meaningful might be problematic. For example: to assume that a missing kinterm means there is no kinterm for that relative. This is because, in the case of secondary sources, it is difficult to know whether a term is absent because a relative is not considered kin, or because it was outside the primary researcher's scope, as the reviewer mentions above. We believe the reviewers comment is in reference to the text found on line 288:

*This presents the difficult challenge of knowing whether a term is absent because this relative is not considered a kin member or because it was not recorded in the source.*

The intention behind this sentence is to relay to the users that we make no inference as to why a kinterm was not recorded for a particular language. But that should not detract users from investigating further. We add the following text after the above sentence on line 290:

*The difference between a language lacking a term for a particular category (true absence), and available records of a language lacking particular terms (data uncertainty), is important. Comparisons with closely related languages, the use of multiple sources, and iterative, community-elicited data collection can help to narrow this uncertainty in future.*

Or that some global analyses might not show any patterns because of "Simpson's paradox" of diverse patterns within different regions?

Kinbank allows researchers to investigate patterns at both the global and regional level. In fact, some researchers have already published on the regional patterns in kinship terminology (Sheard et al., 2020). Simpson’s paradox does indeed apply to cross-cultural analyses, so we would encourage users of the database to explore the relationships between global and regional patterns in kinship terminology and how they may relate to other dimensions of human language or behaviour.

We have added the following text to line 251 highlight this:

*The combination of focused regional samples and broad global coverage will allow future research to compare the patterns and correlates of diversity across different scopes.*

You mention in the supplementary materials that one potential source of disagreement between the different databases is that they relied on different sources. Did you have a criterion to say that you thought a given language is accurately represented or did you simply enter all information you might have found in a representative source? In such cases, did you note that the information might be incomplete? I recognise that you provide details on each entry, but I think it is necessary to provide information on the general rules here in the manuscript.

Data was entered from what we believed to be reliable sources. Coders aimed to, where possible, survey multiple sources on each language. Data entry started with sources with the most comprehensive data and that which covered a majority of terms in G^0^ to G^+1^. We have not noted for each language whether data might be “complete”. As discussed above, from secondary data it is difficult to make such inferences.

You focus on the challenge that occurs when a kin term is absent and you leave a cell empty, but what information is lost in the opposite case where an additional term might exist in a language that encodes a relationship beyond that stored in the 115 types you focus on?

Thank you for pointing this out. This was very briefly mentioned in the main text, but we agree that more detail is useful. With any data collection exercise there are diminishing returns. Our goals were to fill in the set of 155 (see below for the change in total types) kin types as completely as possible for as many languages as possible to provide a wide sample of comparative terminology. In some instances, it was not possible to collect all these terms, but in others there is a long list of kin terms available. So, if a larger set of terms are easily available the new kin types and kin terms are added to the database.

Within the new Collection procedure section above, we have added the following text speaking to this specific point on line 331:

*Some sources (speakers or secondary) may offer many more kinterms that apply beyond the 155 core types. In this instance, we expand the parameter list to accommodate the additional kin terms (see table S2). In total there are 940 kin types within Kinbank, but many of these are sparsely populated. Some languages also contain multiple terms for a particular kin type. In this instance all kin terms are entered separately.*

What was the process through which you came up with the 115 kin types? This seems like it was a critical step in the design of Kinbank, so I think additional information should be provided here. Was this an iterative process? Did you have the genealogical relationships decided a priori? Providing this information will help others to think about what these types might mean, and how they might want to classify relationships

Firstly, upon reflection, a more accurate count of the core kin is 155. The set of 155 kin terms are a combination of consanguineal and affinal terms and were developed to strike a balance between analytical interest (a desire for breadth) and data availability (given our knowledge of extant sources and own resources for the project). We also had accessibility and generalisability in mind: other researchers have made sets of terminologies available using (for example) equivalence statements for terms and using specialist or regional kinship “jargon”, but these are less transparent to students and wider audiences, whom we also consider potential users of the database. The change in core counts occurred because we previously miscounted the core kin types (grandchildren had dropped off the list), and retrospectively ignored the relative age of connecting kin because it was uncommon.

For the set of consanguineal terms, we limited ourselves to two generations above and below the ego (up to grandparents and down to grandchildren). This is a commonly discussed range in the kinship literature, but it also represents the most common set of generations that exist during ego’s lifetime, which is four generations with a generation of between 20-30 years. Not all generations necessarily exist at the same time, e.g., ego might have grandparents when they are young, and grandchildren when they are old. It is uncommon for five generations to co-exist to the point that specific kin terms are needed, although we do include a kin type for an Ancestor kin term (with the parameter A).

From our etically defined genealogical grid, there are four grandparent kin types, four parent’s siblings kintypes, two parents, two siblings, eight cousins, four sibling’s children, two children, and four grandchildren. Within this set we apply three commonly observed rules of distinction: sex of speaker, relative age, and relative age of connecting relative. Sex of speaker applies to all consanguineal kin. Relative age is applied to kin types of the same generation as ego and parent’s siblings. Finally, relative age of connecting kin can only be applied to kin that are connected through multiple kin (e.g., father’s elder brother’s son vs father’s younger brother’s son). We did not systematically search kinterms that combine the rules *relative age* and *relative age of connecting kin*. We never encountered a language which applied both of these rules.

Affinal terms have received significantly less attention than the consanguineal terms, and so our set of terms to look for were based on expert knowledge, rather than any pre-existing standard, as with consanguineal terms. We opted for affinal relationships connected to nuclear family members (e.g., sister’s husband), and nuclear family terms of ego’s affines (e.g., wife’s brother). However, we found that these terms were scarce in the anthropological record. Where we encountered more specific terms (e.g. a special term for mother’s mother’s mother, or for wife’s sister’s brother) we added these to the record for the language they occurred in but do not go back and check what the terms for this are (or if they exist) in every other language in Kinbank, or we would be faced with an infinite regress.

Given that the focus is on the database, maybe some brief descriptive summary of the content could be helpful before you jump into the example analyses. For example, is there any language in which there are separate terms for all these 115 kin types? What is the average number of kin terms per language?

This is an excellent suggestion. We have added the following paragraph to line 374 of the manuscript:

*The Kinbank collection of 1,229 languages offers a large digitised sample of kinship terminology in an accessible format. The collection has 887 languages that contain all grandparent terms, 864 languages with all sibling terms, 728 languages with all terms for parents and parent’s siblings, 604 languages with all nibling terms, and 506 languages with all sibling and cousin terms. The most frequently collected kin terms are all nuclear family terms: father (n = 1,197), mother (1,192), younger brother (1,161), elder brother (1,149), elder sister (1,138), and younger sister (1,134). Closely followed by father’s father (1,110) and mother’s father (1,119). The least common terms, from within the core set, are a combination of affinal and cousin terms: wife’s sister (545), father’s brother’s daughter (552), husband’s sister (552), husband’s brother (554), and mother’s sister’s daughter (556). On average, languages have 23 terms: 2 grandparent terms, 3 sibling terms, 5 terms for parents and parent’s siblings, 4 nibling terms, and 6 sibling and cousin terms. Averages only tell us part of the picture however, as languages can have as few as one kin term for each set (with the exception of parents and parent’s siblings which has a minimum of two) or many terms for every kin type. Although languages range significantly in the number of kin terms they use, some syncretisms are “socially unthinkable” (Héritier, 1981, Godelier 2012). The law of collaterality states that we cannot observe syncretisms between kin that are linked by a member of the opposite sex, without other parallel sycrentisms occurring, a rule which is abided by in all Kinbank languages. For example: Kinbank contains no languages which have a term for father and for mother’s brother, but not father’s brother. Similarly, Kinbank contains no languages that have distinct terms for father’s mother, or mother’s father, with a single term for all other grandparents.*

What does a kappa value of 0.80 signify for inter-rater reliability? Does kappa range between 0-1, with higher values indicating higher agreement? Does this mean that roughly 20% of terms were classified differently between the two raters? Do you think these are systematic biases, where coders primarily familiar with a certain system/group of languages might interpret information differently than coders trained on a different system/language? For the reliability, did you also have native speakers (or linguists) spot-check entries to ensure that the information you transcribed accurately reflects the kin terminology of their language?

You are correct that kappa values range between 0 & 1, and that higher values indicate more agreement. It does not mean that 20% of terms were classified differently. The Kappa value indicates the proportion of the possible agreement beyond random chance. So, the disagreement between coders is likely much lower than 20%.

We did not systematically perform spot checks, but all co-authors checked the languages with which they were familiar. Within the Kinura project, all languages were compiled by an academic expert in Uralic langauges and some languages were input by language specialists. By releasing Kinbank as an open resource we anticipate that other speakers and specialists will help us in the iterative and ongoing process of error correction.

Some more specific question:

Are the emic, language specific, connotations recorded and provided in Kinbank?

Coders included notes for interesting connotation distinctions, and added notes if kin terms did not directly apply to the kin type in question.

Why are Sino-Tibetan languages not included?

Our dataset includes seventeen Sino-Tibetan languages, but so far, we have been unable to enter data for enough languages to replicate the phylogenetically-based sampling used in some other large language families. We are currently seeking further means to help redress this imbalance. Each group had their own collection focus. Varikin would have been the most likely to systematically collect Sino-Tibetan languages, with a focus on languages linked to existing anthropological databases and languages in existing language phylogeny. However, when collection began in 2016, there was no available Sino-Tibetan dated language phylogeny. We are aware that three Sino-Tibetan language phylogenies have been published since, and we hope to add Sino-Tibetan data in the future.

Is there a way for people to contribute?

Yes! This is a core goal of the database which we had neglected to highlight. We have added the following section to line 484:

*Kinbank was designed to be corrected and expanded over time. The ‘living’ datasets are stored at github.com/kinbank. By using Github to store the living datasets, users can raise issues with existing data, propose corrections, and upload new languages. All changes are reviewed by Kinbank authors. New languages can be added by filling in the template document of core kin types found on the homepage of each repository. Living database are not static. To ensure stability to researchers, the datasets are periodically versioned on Zenodo.*

Additional comments:

The second paragraph of the introduction with some examples of kin terminology appears written from an English-speaker perspective, with the assumption that the corresponding kinship terminology will be automatically in the readers mind. As a non-native English speaker, I could follow along, but it might be helpful to phrase the examples in broader context (as you yourself point out in the methods) by always contrasting two or more systems.

We agree it is important to present the examples in the abstract sense to ensure there are no implicit biases. We rephrase the paragraph on line 76 in the following way:

*For example, in Lau Fijian (Fiji) a woman refers to her male sibling using the same word that he refers to her (weka meaning ‘opposite-sex sibling’); Ngiyambaa (Australia) only has one term for children, unlike son and daughter in English; and Dutch (Netherlands) groups parent’s siblings children (cousins), and sibling’s children (nieces and nephews) together under neef (m) and nicht (f).*

To reach the people who apparently have been using outdated concepts based on limited data, it might help to broaden the final paragraph of the introduction by referring what the potential use of the database in different fields of research might be. Currently you focus on specific examples of research that has already been done, which is maybe more limited in scope than what you envision the full potential of the database to be.

We appreciate the thought that our database could influence a wider audience. Kinship is certainly becoming a more central topic of cross-cultural and interdisciplinary work - particularly within historical economics as the first reviewer points out. We add the following text to line 143 to identify more potential uses for this database:

*Kinbank centralises terminology from approximately 15% of known language diversity, at least 3.28 billion speakers (47% of the speakers in Ethnologue), including speakers for 96% of the territories covered in Ethnologue, and at least 20% of every continent (12). Kinbank contains the largest and broadest collection of terminology available, offering a platform to investigate variation in kinship terminology. Researchers can leverage Kinbank to further explore the topics above or many other questions regarding kinship and kinship terminology. We offer two examples of analyses. First, we show a global analysis on forms of kin terms, testing whether we observe gender bias in the phonology of parental terms. Second, we use phylogenetic techniques to test for a co-evolutionary relationship between kinship terminology structure and patterns of cross-cousin marriage. These examples exemplify the types of methodological and theoretical approaches researchers can address using the Kinbank dataset. For example: is there any generalisable relationship between the behavioural structures and social norms of kinship and kinship terminology? What are the unattested categories of kin and what does that tell us about the constraints of language? How learnable are different terminological systems? The questions we can ask of kinship terminology are plentiful. With a comparative database, they are now also testable.*

The examples are also not always immediately accessible to outsiders, for example, it was not clear to me what "a collection of fresh field research alongside new methods of Tupian and Cariban kinship" means and why and for whom this matters. Linked to this, the final sentence of the introduction focuses on "questions on the origin and maintenance of cross-cultural variability in kin categorization", whereas before you seem to have a broader scope in also highlighting studies that investigate how language might guide behavioural norms.

The Tupian-Cariban reference points to a special issue on Tupian and Cariban kinship terminology published by Ciências Humanas at Boletim do Museu Paraense Emílio Goeldi, led by members of the Kinbank team. This sentence is merely pointing to research that was performed with or prompted by the building of this database.

I think it would be helpful if, in the introduction, you have a sentence each about the two example analyses you performed.

We agree with this point and have added the following text on line 145:

*We offer two examples of analyses. First, we show a global analysis on forms of kin terms, testing whether we observe gender bias in the phonology of parental terms. Second, we use phylogenetic techniques to test for a co-evolutionary relationship between kinship terminology structure and patterns of cross-cousin marriage in the Bantu language family.*

Again, given the broad audience this article might reach, I think it could help to provide some brief explanations for some of the analytical choices you made in the two examples. For example, in example 1, why did you use a phylogenetic approach? Naively, the question could be interpreted as a "human bias", so maybe we are simply interested in how many people use these terms? Maybe briefly explain what issue the "regional and historical relationship" introduce such that it is important to "control for linguistic history in comparative hypothesis tests".

We wouldn’t describe example one as a phylogenetic approach. In fact, the primary hypothesis is that the patterns in parental kin terms originate from a non-phylogenetic process. That is, parental terms have their origins in early childhood. Language phylogeny is being used as a control here to avoid the possibility that this pattern would be masked by autocorrelation. Since parental words are considered to be core and early parts of language, these terms will have been passed on and change over time, meaning closely related languages will be similar. If we want to test whether there is a sex bias in parental terms and we do not control for the relationship between languages, then the results will be biased towards any oversampled groups in the dataset. Phylogenetic control allows us to weight each language's parental terms appropriately in the estimation of the relationship between sounds and parental terms.

We have added the following sentence line 611 to make it more clear why phylogeny was included in the sample:

*The regional and historical relationship between [ma] and "father" in Australia highlights the importance of accounting for the interdependence between languages when looking at cross-linguistic patterns. Had the sample been biased towards Australian languages without a historical control, we would conclude that most languages use [ma] for father, but a phylogenetic control accounts for this bias through shared evolutionary history (56)*

In example 1, how many consonants and how many vowels were there? Only the five that are in figure 2? The supplementary material suggests that there are many more. Is the low informativeness of vowels because there are so few of them?

In total there are 33 possible consonant categories in the coding set established by Blasi et al. 2016; we use 30 of these. We decided it was not informative to display all 30 consonants, since many of them are rarely used or have little effect and focused on sounds that were most commonly affiliated with parental terms in the figure based on existing theory.

With regard to vowels, it is unlikely that the reason that they are uninformative is that there are fewer vowel sounds, which we infer that the reviewer means less parameterisation. We stand by our conclusion that vowels are not informative because there is limited sex-bias in the use of vowels, but this conclusion might be more easily justified through the lack of variation in the use of vowels in parental terms. 60% of all parental terms use an [a] vowel sound. Of terms that use an [a] vowel, 34% are mother terms and 25% are father terms. With this knowledge, it is clear that knowing the vowel that is used in a word is unlikely to tell you whether a kin term relates to a mother or father because most of the time, there is only one vowel used. Theoretically, this is expected because [a] sounds are considered maximally distinct from the consonant sounds.

*Reviewer #3*

The paper is well written, presents an important dataset for studies of kinship and cultural evolution, and uses it to assess two important sets of hypotheses. In the future it would be useful to explore reasons for disagreement in the initial reliability testing and perhaps refine the dataset using multiple coders across all languages in the dataset to reduce random and systematic sources of error in the key variables.

We thank the reviewer for their comments. Many of these suggestions were raised above, and we believe we have addressed them there.
